# Correction: Fang et al. Study on Dispersion, Adsorption, and Hydration Effects of Polycarboxylate Superplasticizers with Different Side Chain Structures in Reference Cement and Belite Cement. *Materials* 2023, *16*, 4168

**DOI:** 10.3390/ma17092120

**Published:** 2024-04-30

**Authors:** Yunhui Fang, Xiaofang Zhang, Dongming Yan, Zhijun Lin, Xiuxing Ma, Junying Lai, Yi Liu, Yuliang Ke, Zhanhua Chen, Zhaopeng Wang

**Affiliations:** 1 Polytechnic Institute, Zhejiang University, Hangzhou 310015, China; fangyunhui@126.com; 2 College of Civil Engineering and Architecture, Zhejiang University, Hangzhou 310058, China; dmyan@zju.edu.cn; 3 KZJ New Materials Group Co., Ltd., Xiamen 361101, China; 15859120891@163.com (X.Z.); geniusvcap@163.com (Z.L.); mary@xmabr-kzj.com (X.M.); yuliang_ke8207@163.com (Y.K.); zhanhuachen123@126.com (Z.C.); wangzp1109@126.com (Z.W.); 4 School of Materials Science and Engineering, Zhejiang University, Hangzhou 310023, China; liuyimse@zju.edu.cn

**Error in Table**

In the original publication [1], there were some minor mistakes in the Tables 1, 3, 6 and 8 because of the authors’ negligence. The correct Table 1, Table 3, Table 6 and Table 8 appear below:

**Table 1:** Chemical composition of the cement (wt%).

Cement	CaO	SiO_2_	Al_2_O_3_	Fe_2_O_3_	MgO	SO_3_	Na_2_O	K_2_O	MnO	TiO_2_	LOI *
RC	63.79	19.80	5.12	3.65	2.30	2.49	0.30	0.31	0.12	0.16	1.85
LC	58.98	21.72	5.60	3.53	2.55	2.10	0.31	0.38	0.15	0.14	4.00

* Loss of ignition.

**Table 3:** Mineral compositions of the cement (wt%).

Cement	C_3_S	C_2_S	C_3_A	C_4_AF	CaSO_4_
RC	62.78	9.40	7.60	7.25	4.23
LC	26.49	42.28	8.87	7.02	3.57

**Table 6:** Fitting results of Bingham, Herschel–Bulkley, and modified Bingham models for the rheological curves of different PCE in two types of cement slurry.

Sample	Bingham			Herschel–Bulkley				Modified Bingham			
	τ/ mPa	η/ (mPa·s)	R^2^	K/ (Pa·sn)	τ/ mPa	n	R^2^	μ	τ/ mPa	a	R^2^
RC-PC-1	11.173	1.8547	0.9995	3.185	2.793	0.836	0.998	1.602	10.060	−0.0017	0.9974
RC-PC-2	15.477	1.3696	0.9957	1.766	12.008	1.010	0.999	1.807	12.285	0.0004	0.9995
LC-PC-1	2.153	1.0020	0.9948	3.290	−7.503	0.715	0.999	1.028	−0.075	−0.0021	0.9994
LC-PC-2	6.571	0.7431	0.9900	0.586	6.684	1.106	0.995	0.834	6.057	0.0012	0.9964

**Table 8:** Parameters of hydration heat-release curves of different PCEs in two types of cement.

Sample	t_0_ (h)	q_0_ (mW/g)	Q_0_ (J/g)	t_2_ (h)	K_2_ (mW/(g∙h))	q_2_ (mW/g)	t_3_ (h)	q_3_ (mW/g)	Q_3_ (J/g)	Q_0–3_ (J/g)
RC-Blank	1.68	0.36	2.92	5.60	0.38	1.38	12.15	2.77	80.41	77.49
LC-Blank	2.15	0.24	3.10	6.70	0.49	1.60	12.36	3.38	92.85	89.75
RC-PC-1	4.33	0.28	7.15	10.37	0.33	1.39	15.77	2.68	94.59	87.44
RC-PC-2	3.21	0.30	3.50	13.95	0.34	1.56	18.59	2.67	99.56	95.78
LC-PC-1	2.90	0.13	1.46	12.12	0.51	1.65	16.93	3.55	97.81	96.35
LC-PC-2	2.16	0.24	1.81	15.40	0.43	1.73	19.60	3.19	109.27	107.46

There was a mistake in Table 10 as published. Four images were selected incorrectly, the time information was deleted from the SEM images, and the scale bars were consistently retained. The corrected Table 10 appears below:

**Table 10:** Morphological analysis of hydration products of RC and LC with different water reducers at 12 h and 3 d.

Cement	Hydration Time	Blank	PC-1	PC-2
RC	12 h	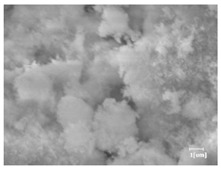	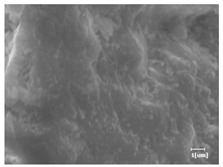	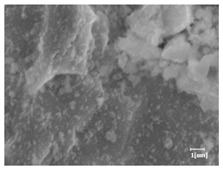
LC		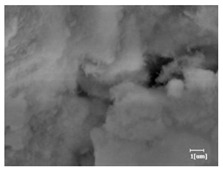	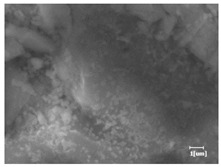	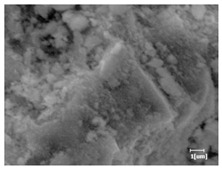
RC	1 d	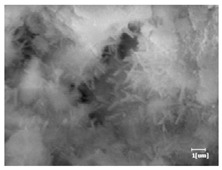	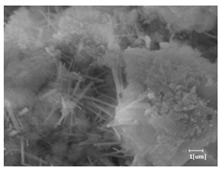	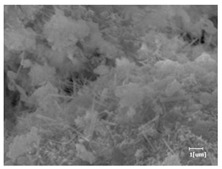
LC		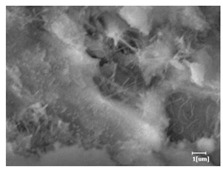	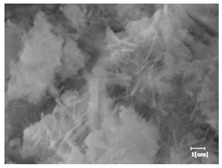	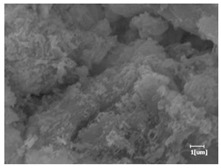
RC	3 d	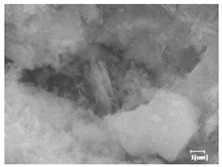	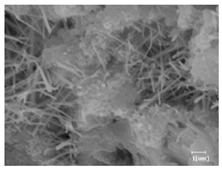	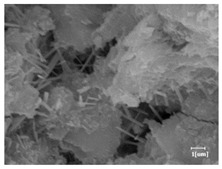
LC		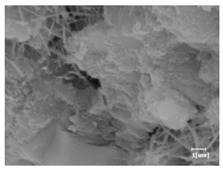	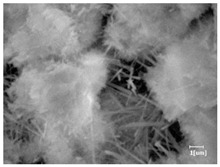	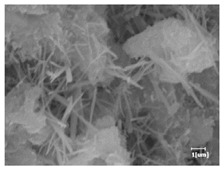

**Error in Figure**

In the original publication [1], there were some minor mistakes in Figures 5, 7a, and 13 as published because of the authors’ negligence. The corrected Figure 5, Figure 7a, and Figure 13 appear below:

**Figure 5:**
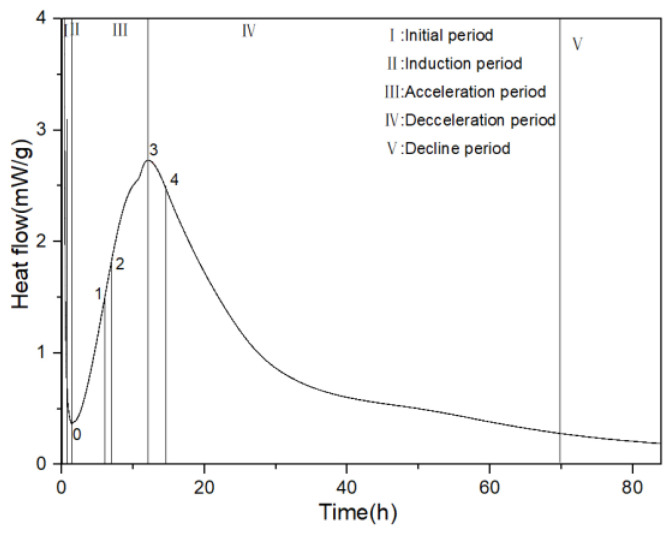
Five stages of hydration heat release.

**Figure 7:**
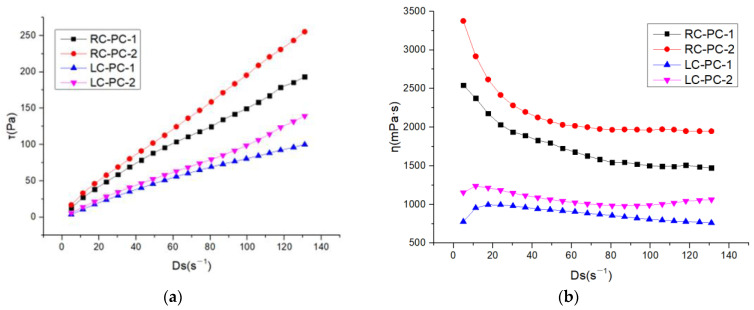
Rheological properties of different water reducers in two types of cement slurries: (**a**) shear rate–shear stress; (**b**) shear rate–apparent viscosity.

**Figure 13:**
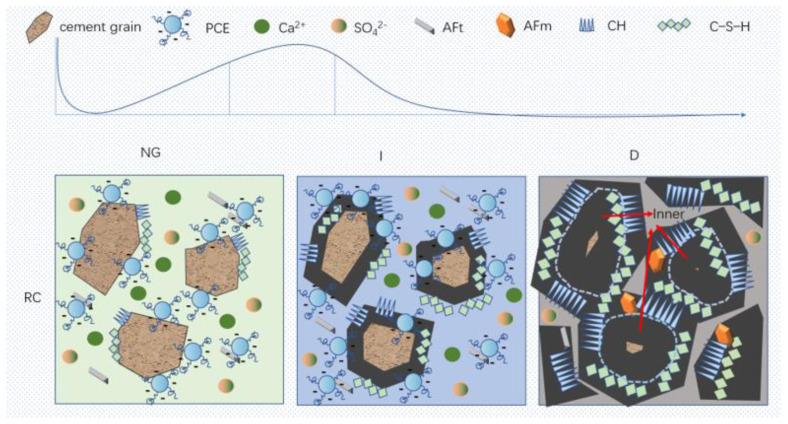

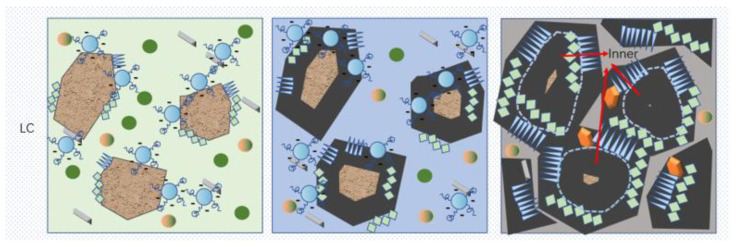
Schematic diagram of the hydration mechanism.

**Text Correction**

In the original publication [1], there were some mistakes because of the authors’ negligence. 

A correction has been made to Section 2.2.1. Gel Permeation Chromatography, Paragraph 1:

The temperature was maintained at 25 °C, and a 0.1 mol/L NaNO_3_ aqueous solution with a pH of 7 was used as the eluent, with dextran of different molecular weights as the calibration standards. PCE was diluted to 5 mg/mL with a 0.1 mol/L sodium nitrate solution. GPC was performed using a Waters 1515 instrument (Waters, Milford, MA, USA) equipped with a differential refractive index detector. Additionally, a multi-detection system (Malvern Viscotek 270 Dual Detector) equipped with viscosity and low-angle laser light-scattering detectors was utilized.

A correction has been made to Section 2.1.1. Cement, Paragraph 3:

The content of C_3_S, C_2_S, C_3_A, C_4_AF, and CaSO_4_ in the two types of cement was calculated based on the data in Table 1, and the results are presented in Table 3. A comparison of the mineral compositions of the two types of cement reveals that the content of C_3_S, C_4_AF, and CaSO_4_ in the RC is higher by 36.3%, 0.23%, and 0.66%, respectively, while the C_2_S content in the RC is lower by 33.9% compared to that in the LC.

The authors state that the scientific conclusions are unaffected. All the above corrections were approved by the Academic Editor. The original publication has also been updated.
